# The Interconnection between Carotid Intima–Media Thickness and Obesity: Anthropometric, Clinical and Biochemical Correlations

**DOI:** 10.3390/medicina59091512

**Published:** 2023-08-23

**Authors:** Danira Bažadona, Martina Matovinović, Magdalena Krbot Skorić, Hrvoje Grbavac, Andrej Belančić, Branko Malojčić

**Affiliations:** 1 Department of Neurology, University Hospital Centre Zagreb, 10000 Zagreb, Croatia; b.danira@gmail.com (D.B.); mkrbot@gmail.com (M.K.S.); 2 Croatian Referral Center for Obesity Treatment, Department of Endocrinology, University Hospital Centre Zagreb, 10000 Zagreb, Croatia; martina_10000@yahoo.com; 3 University Psychiatric Hospital Vrapce, 10090 Zagreb, Croatia; hgrbavac@gmail.com; 4 Department of Clinical Pharmacology, Clinical Hospital Centre Rijeka, 51000 Rijeka, Croatia; a.belancic93@gmail.com; 5 Department of Basic and Clinical Pharmacology with Toxicology, Faculty of Medicine, University of Rijeka, 51000 Rijeka, Croatia

**Keywords:** carotid intima–media thickness, obesity, vascular age

## Abstract

*Background and Objectives*: Carotid intima–media thickness (CIMT) and obesity are considered independent determinants of cardio- and cerebrovascular events. The aim of our study was to investigate the effect of obesity on CIMT and to define which traditional cardiovascular risk factors correlate the most with CIMT values in patients with obesity. *Materials and Methods*: Anthropometric measurements were collected for the whole study group, as well as body composition and blood pressure data, and biochemical blood analyses were also performed. *Results*: Although our study group was significantly older according to vascular compared with chronological age, the mean CIMT values were lower when compared with the reference values. We found a statistically significant correlation of CIMT with chronological and vascular age, systolic blood pressure, fasting glucose, total cholesterol and triglyceride levels, waist-to-hip ratio, waist circumference, body muscle mass and skeletal muscle mass index. Atherosclerotic Cardiovascular Disease (ASCVD) risk assessment and SCORE (Systematic COronary Risk Evaluation) showed significant positive correlations, but there was only a weak correlation of ASCVD with CIMT. *Conclusions*: To deduce, since no diagnostic tool currently includes body weight as an individual risk factor, further trials are highly needed to determine if SCORE, SCORE2, ASCVD risk assessment or CIMT would be the most accurate and relevant diagnostic tool for prediction of risk for future CV events in patients with obesity.

## 1. Introduction

Carotid intima–media thickness (CIMT) and obesity are considered independent determinants of cardio- and cerebrovascular events. Obesity is associated with increased cardiovascular disease in primary prevention, but in secondary prevention, mortality is often lower in people with normal body weight [1]. The association of traditional cardiovascular risk factors, including obesity, with CIMT still remains unclear. Some studies have shown a clear effect of obesity on CIMT, with not only body mass index (BMI), but even other anthropometric measurements showing a greater impact [2,3,4]. The potential of CIMT measurement for the early detection of vascular remodeling was also demonstrated by the research of Lucas-Herald et al. in a younger population [5]. CIMT was shown to be a convenient method for observing the reversibility of arterial wall thickness in individuals with obesity after the change in various variable factors, such as diet, nutrient intake, and weight loss [6]. Also, CIMT appears to be a simple method that can monitor the transition of a person with normal body mass to obesity as well as to a metabolically abnormal status [7]. The current body of literature is still generally inconclusive, although one interesting study showed no difference in CIMT between men and women with obesity after adjustment for classic cardiovascular risk factors, while premenopausal status was protective; thus, more well-designed studies are needed [8]. Different methods can be utilized as indicators of the risk of cardiovascular diseases such as CIMT, pulse wave velocity (PWV) and biomarkers of endothelial dysfunction, but people with body mass index (BMI) grade III had significantly higher levels of PWV and CIMT than the control group [9]. The goal of our study was to determine the effect of obesity on CIMT in people with no previous myocardial infarction, stroke or transient ischemic attack (TIA), using diverse anthropometric parameters. Also, we aimed to determine which traditional cardiovascular risk factors are most closely correlated with CIMT values in patients with obesity.

## 2. Materials and Methods

This cross-sectional study was conducted at the Croatian Referral Center for Obesity Treatment (which is also and EASO collaborating center for obesity management) and at the TIA Clinic of the University Hospital Center Zagreb, Croatia. The study was approved by the hospital’s Ethics Committee. All the participants signed an informed consent form. Overall, the study was conducted in accordance with the Declaration of Helsinki.

We included 101 patients with obesity of both genders. The inclusion criteria were BMI ≥ 30 kg/m^2^ and age > 18 years. The exclusion criteria were pituitary and/or adrenal disease, untreated thyroid disease, prior myocardial infarction, stroke, TIA or oncological disease. Patients’ weight (kg), height (m), hip (cm), waist (WC; cutoffs proposed by WHO for increased metabolic complication risk are 94 cm in European men and 80 cm in women, and 102 and 88 cm for substantially increased risk, respectively) (cm) and neck circumference (NC; cutoffs proposed are 37 cm in men and 34 cm in women) (cm) were measured; body mass index (BMI) (weight/height^2^), waist-to-hip ratio (WHR; cutoffs proposed by WHO for substantially increased metabolic complication risk are ≥0.90 in men and ≥0.85 in women) were calculated [10,11]. Body composition was estimated using bioelectrical impedance analysis using body composition analyzer model Tanita SC-330 (Tanita Europe B:V: 2004). Patients’ body fat percentage, body fat and muscle mass (kg) were measured to calculate skeletal muscle mass index (SMI) (kg/m^2^) and fat mass index (FMI) (kg/m^2^). The information on smoking status (non-smoker and current smoker), diabetes, hypertension, number of years taking antihypertensive drugs and number of years living with obesity was recorded for each patient. 

Blood pressure was measured with an OMRON digital sphygmomanometer on both arms after at least 10 min of rest. Mean values were determined from two independent measurements. Patients with systolic blood pressure (SBP) below 140 mmHg and diastolic blood pressure (DBP) below 90 mmHg were considered normotensive. Patients with average blood pressure above 140/90 mmHg or those who took antihypertensive medication were put in the hypertensive group. Vascular age was determined by using color cardiovascular risk scales from the SCORE (Systematic Coronary Risk Evaluation project) [12]. Atherosclerotic Cardiovascular Disease (ASCVD) risk was calculated according to the AHA/ACC guidelines [13].

The biochemical analyses in all patients included fasting glucose, fasting insulin, plasma glucose 2 h after a 75 g oral glucose load, total cholesterol, LDL and HDL cholesterol, triglycerides and thyrotropin. Thyrotropin was obtained with ECLIA Roche (analyzer Cobas e6000). Plasma glucose was measured by a glucose-oxidase method. Plasma total cholesterol, HDL cholesterol, and triglycerides were assessed with standard enzymatic spectrophotometric techniques. Plasma insulin was measured by radioimmunoassay. HOMA-IR was calculated with the formula: fasting plasma glucose (mmol/L) times fasting serum insulin (mIU/L) divided by 22.5, according to the method previously described and standardized elsewhere [14].

CIMT was measured according to the Mannheim protocol using a linear ultrasound transducer (3–12 MHz) [15]. Patients were in a supine position with their neck in extension and the probe was in an antero-lateral position. All measurements were performed in longitudinal view on the far wall of the common carotid artery (CCA). The measurements on the right and the left CCA were performed twice, and the mean values were used for analysis. All patients were examined on the same ultrasound system (Affiniti 70 with a linear 4–12 MHz probe from Philips Ultrasound Inc., Amsterdam, The Netherlands), using automated edge detection software (Philips QLAB 10.8.20). For every participant, age- and gender-specific reference CIMT values (meanCIMTage and gender, and standard deviation age and gender) were calculated according to equations for CIMT for the healthy population [16]. Based on these calculations, an age- and gender-specific z-score was calculated for each participant ((mean CIMT-meanCIMTage and gender)/standard deviation age and gender).

Statistical analysis was performed using SPSS 25 software (IBM, Armonk, NY, USA). To assess the distribution of the data, the Kolmogorov–Smirnov test was applied. Variable values were described by absolute and relative frequencies, central tendency measures, and spread measures. Associations between variables were tested with Pearson’s (correlation coefficient, r_p_) and Spearman’s (correlation coefficient, r_s_) correlation methods for parametric and non-parametric distributions, respectively. For the categorical variables, the differences were determined with the Chi-square test. Differences between two groups were determined with the parametric *t*-test or non-parametric Mann–Whitney test, whilst for comparison between three or more groups, an ANOVA test was used. Univariable linear regression analysis was performed to examine which demographic and clinical variables were significant predictors for the mean CIMT. Variables for analysis were included based on clinical significance. Statistically significant predictors were included in the multivariable linear regression model. *p* values of less than 0.05 were considered significant. 

## 3. Results

### 3.1. CIMT Values

The study included a total of 101 patients with obesity, 75 (74.3%) of whom were female. The main characteristics of the study group are presented in Table 1. The mean values of right and left CIMT were 0.576 ± 0.133 (range 0.32 to 0.97) mm and 0.589 ± 0.130 (range 0.31 to 1.13) mm, respectively. Since there were no statistically significant differences between the measurements of the right and left CIMT (*p* = 0.166), the mean CIMT value was calculated and used in further statistical analysis. The average mean CIMT value was 0.583 ± 0.122 (range 0.315–1.050) mm. There was no statistically significant difference in mean CIMT values between a male and female group of patients (0.594 ± 0.107 vs. 0.579 ± 0.127, *p* = 0.603).

### 3.2. Age and CIMT

The mean age of the study group was 49.02 ± 12.52 years, with the youngest being 22 and the oldest 78 years. Vascular age was calculated according to the cardiovascular risk scales from the SCORE project [12]. Due to some missing data, we were able to calculate vascular age for only 71 patients. The mean vascular age was 54.32 ± 9.9 years, with a range from 39 to 74 years. The study cohort was significantly older according to vascular age in comparison with chronological age (54.32 ± 9.9 vs. 51.94 ± 8.2, *p* < 0.001; confined to patients who had the vascular age calculated). 

**Figure 1:**
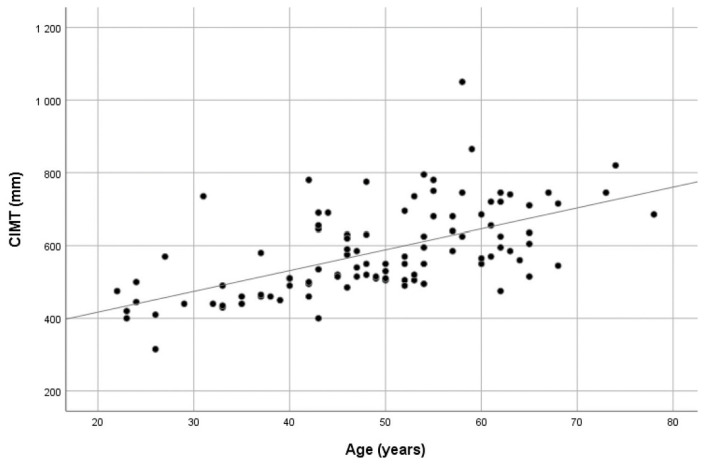
Scatter plot showing the distribution of CIMT values over chronological age.

**Figure 2:**
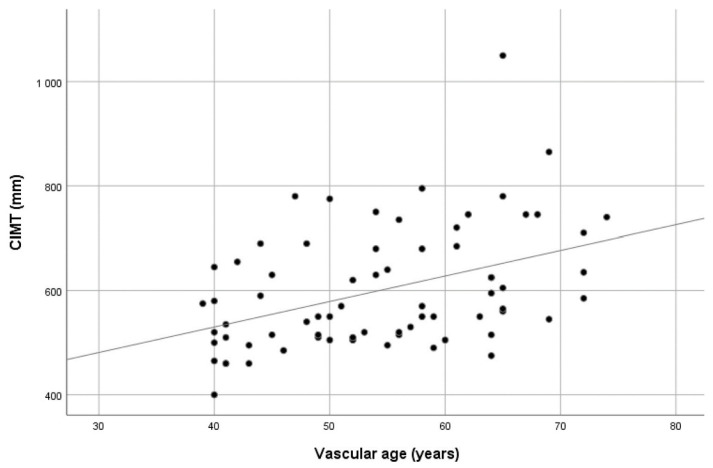
Scatter plot showing the distribution of CIMT values over vascular age.

The mean CIMT values showed a significant positive correlation with both chronological and vascular age (r_p_ = 0.588 and r_p_ = 0.421, respectively, all *p* values < 0.01). Scatter plots showing the CIMT values dispersion over age are presented in Figure 1 and Figure 2. When the study group was divided according to vascular age, i.e., those with higher vascular age values in comparison with chronological age (60.6% of patients) and those with corresponding or lower vascular age values (39.4% of patients), no significant difference in CIMT was found (0.614 ± 0.129 vs. 0.578 ± 0.087, *p* = 0.197). See Supplementary Table S1.

### 3.3. Anthropometric Measurements and CIMT

**Figure 3:**
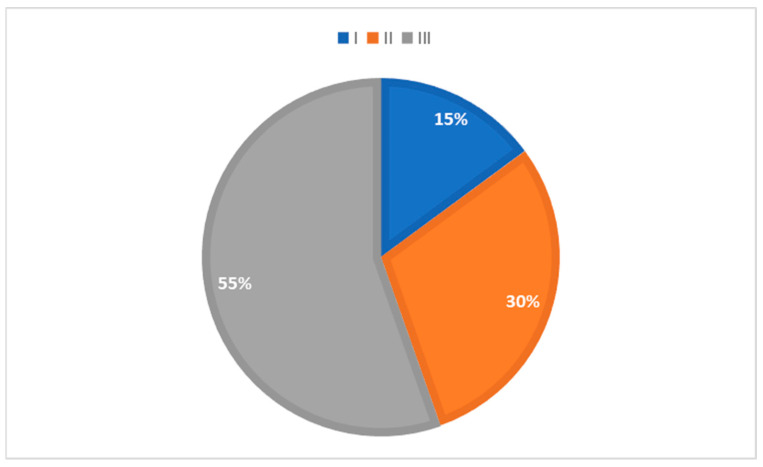
Patients divided according to obesity grade (I—BMI 30.0–34.9, II—BMI 35.0–39.9 and III—BMI above 40).

The percentage of patients in groups divided according to the obesity grade is presented in Figure 3. No statistically significant difference was found in mean CIMT values when comparing the groups divided according to their BMI grade (F(2.98) = 0.967; *p* = 0.384). See Supplementary Table S2. In 82.2% and 84.7% of patients, neck circumference and WHR values were higher than the set cut-off [17,18]. 

**Figure 4:**
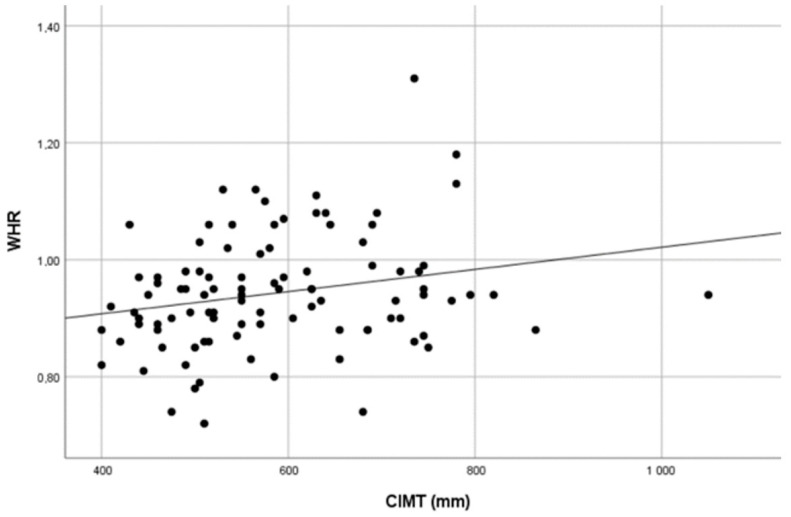
Scatter plot showing the distribution of CIMT values over WHR values.

We found statistically significant positive correlations between mean CIMT values and body muscle mass, SMI, waist circumference and WHR. A Scatter plot showing the distribution of CIMT values over WHR values is presented in Figure 4. No statistically significant correlations between mean CIMT and BMI, body fat percentage, weight, body muscle percentage, FMI or neck and hip circumference were found. The results are presented in Table 2. 

### 3.4. The Effects of Traditional Cardiovascular Risk Factors on CIMT

Arterial hypertension was present in 59.4% of patients, with a mean duration of 7.18 ± 5.88 years. There was a statistically significant difference in mean CIMT values between groups with and without arterial hypertension (median 0.585 vs. 0.500, *p* < 0.001), whereby patients with hypertension had higher values of mean CIMT. The values of systolic blood pressure showed a significant positive correlation with mean CIMT values, although the diastolic blood pressure and the duration of arterial hypertension did not show any effect on CIMT. 

In 15.8% of patients, diabetes mellitus was present, with a median duration of 1 year (range: 0.6 to 10 years), while 30.7% had prediabetes defined according to the American Diabetes Association [19]. The median value of HOMA-IR was 3.760 (range: 0.810 to 21.950). There was no statistically significant difference in mean CIMT between the group with diabetes and the group with no diabetes (0.634 ± 0.086 vs. 0.573 ± 0.125, *p* = 0.066), or between the groups with and without prediabetes (0.619 ± 0.131 vs. 0.567 ± 0.115, *p* = 0.051). No statistically significant correlation was noted between the duration of diabetes and mean CIMT. The mean CIMT values showed a statistically significant positive correlation with fasting glucose levels, but not with glucose levels after two hours in OGTT, insulin levels or HOMA-IR. 

Dyslipidaemia was present in 42.4% of patients, but the groups divided according to the presence of dyslipidemia, or according to the use of statins did not show statistically significant differences in mean CIMT values (0.605 ± 0.122 vs. 0.565 ± 0.121, *p* = 0.104 and 0.606 ± 0.090 vs. 0.580 ± 0.126, *p* = 0.474, respectively). When looking independently at levels of total cholesterol, HDL, LDL and triglycerides, mean CIMT significantly correlated with total cholesterol and triglyceride levels. 

Among the whole group, 22.1% were current smokers. The comparison between the smoking and non-smoking groups showed no difference in mean CIMT values (0.598 ± 0.137 vs. 0.584 ± 0.118, *p* = 0.630). 

A weak correlation between the patients’ ASCVD risk and CIMT values was found, while there was a significant positive correlation between ASCVD risk and SCORE (r = 0.313, *p* = 0.010 vs. r = 0.629, *p* < 0.01).

### 3.5. Univariable and Multivariable Regression Analysis of Possible CIMT Predictors

Clinically significant variables were included in a regression analysis in order to determine the possible predictors for mean CIMT values. The results of the univariable linear regression are presented in Table 3. According to this analysis, chronological and vascular age, arterial hypertension, systolic blood pressure and WHR were positive predictors of higher CIMT values. These variables were included in the multivariable regression model together with age and gender. The results are presented in Table 4. After correction for age, only WHR remained a statistically significant positive predictor for the mean CIMT value. 

### 3.6. CIMT Z-Score Analysis

Based on the previously published equations, we calculated expected normal mean CIMT values for every patient, which were then used for determination of age- and gender-specific CIMT Z-scores [16]. Across the group, the mean CIMT Z-score was 0.144 ± 1.020 (range from −1.781 to 4.297), with a median value of −0.197. The results of univariable linear regression analysis indicating which parameters are possible significant predictors for CIMT Z-scores are presented in Table 5. WHR was a positive predictor of a higher CIMT Z-score.

## 4. Discussion

The most interesting result is that our study group, although obese and significantly older according to SCORE, had lower mean CIMT values compared with the reference values. Considering the fact that our study was conducted without age- and gender-matched control subjects, the normal, expected CIMT values were calculated according to the previously mentioned mathematical formula [16]. Compared with those reference values, our cohort did not show a deviation as expected. On the contrary, the median results of the CCA Z-scores were even lower than the reference values. When the CIMT of each patient was matched with its reference value, our adipose cohort had CIMT values as thin as the non-adipose, healthy population. 

The other interesting finding was a significant positive correlation between the patient’s ASCVD risk and SCORE, but only a weak correlation of ASCVD with CIMT. This finding confirms the consistency of results in our group, i.e., patients with higher vascular age also had a higher risk for cardiovascular diseases, but not a higher CIMT. 

### 4.1. The Effect of Anthropometric Measurements on CIMT

When evaluating the impact of different adiposity parameters on CIMT, a positive correlation with hip circumference and WHR was found. No difference in CIMT was observed when the group was divided according to their BMI values. As expected, WHR as a measure of central obesity showed a greater correlation with CIMT than BMI. These results are in concordance with some of the previously published results in which anthropometric parameters showed a greater impact on CIMT than the metabolic parameters [20]. In the univariate regression analysis, WHR was the only positive predictor of higher CIMT Z-score, while systolic and diastolic blood pressure showed a trend toward significance. In the SABPA study, Veldsman et al. showed significant positive relationships between CIMT and WC in teachers undertaking light physical activity [21]. 

According to some previous studies, lean body mass, and not BMI or fat mass, independently contributes to CIMT in middle age subjects [22]. In a study conducted on a large cohort of healthy young people, the increase in CIMT was associated with an increase in lean body mass and systolic blood pressure, whereas it correlated negatively with body fat mass [23]. These findings support the hypothesis that the increase in cardiac output and systolic blood pressure causes arterial remodeling as an adaptation to luminal hemodynamics. The main effect on the carotid wall could be medial, but not intimal thickening [24]. Another large study on lean body mass effect on CIMT showed similar results, with other body size and adiposity measurements (e.g., waist circumference and waist-to-hip ratio) having an effect on CIMT, but not on carotid plaque burden [25]. According to those results, the associations of adiposity and CIMT should not be interpreted as reflecting the effect of adiposity on atherosclerosis [24]. We found statistically significant positive correlations between mean CIMT values, body muscle mass and SMI, which is consistent with the aforementioned studies [22,23]. 

### 4.2. The Effect of Age on CIMT

Among the non-modifiable risk factors for cardiovascular diseases, age showed the most consistent effect on CIMT. The progression of CIMT with aging was shown to be 0.052 mm per year in men and 0.050 mm in women [16]. In our study, besides chronological age, we also used vascular age as calculated according to scales for low-risk countries from the SCORE study [12]. 

Our group had significantly higher vascular than chronological age, meaning that their vessels are older than expected. Both chronological and vascular age were shown to be strong determinants of CIMT. There was no difference in CIMT values when the whole study group was divided according to their vascular age, i.e., those with higher and those matching their chronological age. In the univariate linear regression analysis, both chronological and vascular age were positive predictors of higher CIMT values. 

### 4.3. The Effect of Traditional Cardiovascular Risk Factors on CIMT

There was a clear difference in CIMT between the patients with and without arterial hypertension, regardless of its duration, with the hypertensive group having higher CIMT values. Systolic, but not diastolic blood pressure, was shown to positively correlate with CIMT, which is in line with the results of previously published studies [26,27,28]. Similarly, results of a population-based cohort study showed that systolic BP is linearly associated with higher CIMT in both hypertensive and nonhypertensive persons [28]. 

Neuhauser et al. provide strong evidence of an association between hypertension, obesity, and increased CIMT in young people, adjusted for height, age, and gender [29].

CIMT values were shown to be higher in diabetic and prediabetic patients [2,30,31,32]. Studies showed that in prediabetes, both impaired glucose tolerance and impaired fasting glucose were associated with increased CIMT [33,34]. We found a significant positive correlation between CIMT and fasting glucose levels, although no difference in CIMT between patients with diabetes or prediabetes and without diabetes or prediabetes was seen. 

There was no difference in CIMT between patients with and without dyslipidemia, which could be explained by the fact that one third of them were on lipid-lowering drugs. This is supported by the fact that CIMT had a significant positive correlation with total cholesterol and triglyceride levels. The Multi-Ethnic Study of Atherosclerosis showed that hyperlipidemia and hypercholesterolemia were associated with increased CIMT, but in populations without diabetes mellitus and lipid-lowering therapy [35].

One of the possible explanations for lower mean CIMT values in our study group is that we excluded patients with previous myocardial infarction, stroke, or TIA; additionally, hypertension and diabetes were diagnosed and treated in a timely manner so the impact of these risk factors on CIMT was smaller. The fact that the thickness of CIMT did not depend on the duration of hypertension and diabetes supports this explanation. A meta-analysis of 22 trials also showed a beneficial effect of antihypertensive treatment on CIMT [36]. 

Further insights have been obtained by observing the intima and media thickness separately with high-frequency ultrasound [24]. When measuring intimal and medial thickness and their ratio, a significant difference was found between subjects with and without cardiovascular diseases, but the difference was lost when observing intima–media thickness as one [25]. It was shown that the thickness of the intimal layer increases, while the thickness of the media decreases with increasing age and the degree of atherosclerosis [24]. The thickness of the intima correlates with traditional cardiovascular risk factors [37]. As there are two different processes occurring in arteries—intimal thickening as a result of atherosclerosis, and medial thickening as a sign of arterial remodeling—the measurements of each layer individually could lead to a better perception of changes in the arterial wall and consequently better risk stratification of patients. This could help resolve the inconsistency of results across different studies evaluating the correlation of CIMT and traditional cardiovascular risk factors. 

The strength of this study is that it included different risk calculations and that most of the patients were in the BMI class III group, which we know is a very rare prevalence in Europe, [38], although we do not know the prevalence in Croatia. In the present study, the risk was calculated as per SCORE to enable inclusion of the parameter of vascular age; however, it would be interesting in the future to reproduce this methodology with SCORE 2. 

## 5. Conclusions

We demonstrated the correlation of CIMT with several clinical, biochemical and anthropometric cardiovascular risk determinants, such as age, arterial hypertension status (especially SBP), fasting glucose, total cholesterol, triglycerides, WHR, waist circumference, body muscle mass and SMI, which is consistent with the currently available relevant body of literature. Although our study group was significantly older according to vascular age relative to chronological age, no statistically significant difference in CIMT was noted when the mean measured values were compared with the expected normal age- and gender-adjusted values. The ASCVD risk assessment showed a significant positive correlation between the patients’ ASCVD risk and SCORE, but only a weak correlation of ASCVD with CIMT.

Whether increased CIMT in the different populations investigated is due to intimal or medial thickening remains an open question. The answer can probably be determined with more sophisticated and detailed techniques, such as high-frequency ultrasound, which could be used to determine the exact pattern of changes in the arterial wall. 

In conclusion, since no diagnostic tool currently includes body weight as an individual risk factor, further trials are highly necessary to determine if SCORE, SCORE2, ASCVD risk assessment or CIMT would be the most accurate and relevant diagnostic tool for the prediction of risk for future CV events in patients with obesity because none of these diagnostic tools include body weight as a risk factor.

## Figures and Tables

**Table 1:** The main study group characteristics.

Variable	Number	Mean ± St. Dev.	Median (Min–Max)
*Anthropometric measurements*			
BMI (kg/m^2^)	101	43.47 ± 9.4	
Weight (kg)	101	124.16 ± 31	
Height (m)	101	1.685 ± 0.091	
Body fat percentage (%)	98	45.74 ± 7.13	
Body fat mass (kg)	98	56.24 ± 17.33	
FMI (kg/m^2^)	98	19.79 ± 5.52	
Body muscle percentage (%)	98	51 ± 8	
Body muscle mass (kg)	98		58.25 (10.0–138.6)
SMI (kg/m^2^)	98		20.82 (2.89–44.24)
Neck circumference (cm)	101	41.14 ± 4.89	
Waist circumference (cm)	100	125.29 ± 20.41	
Hip circumference (cm)	98	133.63 ± 14.95	
WHR	98	0.94 ± 0.1	
*Biochemical analysis*			
Fasting glucose (mmol/L)	101	5.57 ± 0.99	
Plasma glucose 2 h after a 75 g oral glucose load (mmol/L)	81	5.54 ± 1.64	
Fasting insulin (mIU/L)	97		16 (3.8–88.2)
HOMA-IR	98		3.77 (0.81–21.95)
Total cholesterol	93	4.93 ± 1.08	
LDL (mmol/L)	95	2.92 ± 0.88	
HDL (mmol/L)	95	1.26 ± 0.32	
Triglycerides (mmol/L)	95		1.3 (0.39–6.03)
*Comorbidities*			
Obesity duration (years)	101		12 (1–35)
Type 2 diabetes mellitus (%)	15.8		
Arterial hypertension (%)	59.4		
Systolic blood pressure (mmHg)	100	131 ± 17	
Diastolic blood pressure (mmHg)	100	82 ± 13	
Dyslipidemia (%)	42.4		
*Medication*			
Antihypertensive medication (%)	55.4		
Anti-diabetic medication (%)	14.9		
Statins (%)	12.9		

Descriptive values of measured parameters: For variables with parametric distributions, the mean and St. Dev. are shown. For variables with non-parametric distributions, the median, min and max are shown. All participants had thyrotropin values within the reference range (0.35–4.90 mIU/L). BMI (body mass index), FMI (fat mass index), SMI (skeletal muscle mass index), WHR (waist-to-hip ratio), HOMA (homeostatic model assessment for insulin resistance), LDL (low-density lipoprotein), HDL (high-density lipoprotein), ASCVD (Atherosclerotic Cardiovascular Disease).

**Table 2:** Correlation of mean CIMT with analyzed parameters.

Correlation of Mean CIMT with:	r, *p*
*Age*	
Chronological age	**r = 0.588, *p* < 0.01**
Vascular age	**r = 0.421, *p* < 0.01**
*Anthropometric measurements*	
BMI	r = 0.028, *p* = 0.782
Body fat percentage	r = −0.136, *p* = 0.183
Body fat mass	r = 0.007, *p* = 0.943
FMI	r = −0.030, *p* = 0.773
Body muscle percentage	r = 0.106, *p* = 0.297
Body muscle mass (BMM)	**r = 0.217, *p* = 0.032**
SMI	**r = 0.259, *p* = 0.01**
Neck circumference	r = 0.184, *p* = 0.065
Waist circumference	**r = 0.235, *p* = 0.019**
Hip circumference	r = 0.063, *p* = 0.537
WHR	**r = 0.232, *p* = 0.022**
*Biochemical analysis*	
Fasting glucose	r = 0.299, *p* = 0.002
Plasma glucose 2 h after a 75 g oral glucose load	r = 0.104, *p* = 0.355
Fasting insulin	r = 0.062, *p* = 0.546
HOMA-IR	r = 0.102, *p* = 0.316
Total cholesterol	**r = 0.209, *p* = 0.044**
LDL	r = 0.166, *p* = 0.108
HDL	r = −0.015, *p* = 0.886
Triglycerides	**r = 0.259, *p* = 0.011**
*Comorbidities*	
Obesity duration	r = 0.172, *p* = 0.085
Systolic blood pressure	**r = 0.273, *p* = 0.006**
Diastolic blood pressure	r = 0.076, *p* = 0.453
Arterial hypertension duration	r = 0.188, *p* = 0.151
Type 2 diabetes mellitus duration	**r = −0.361, *p* = 0.170**
ASCVD risk	r = 0.313, *p* = 0.010

Statistically significant values are highlighted. BMI (body mass index), FMI (fat mass index), SMI (skeletal muscle mass index), WHR (waist-to-hip ratio), HOMA (homeostatic model assessment for insulin resistance), LDL (low-density lipoprotein), HDL (high-density lipoprotein), ASCVD (Atherosclerotic Cardiovascular Disease).

**Table 3:** Univariable linear regression analysis of possible CIMT predictors.

	B, 95% C.I. for B	*p* Value
Chronological age	**B = 0.006, 0.004–0.007**	***p* < 0.01**
Vascular age	**B = 0.005, 0.002–0.007**	***p* < 0.01**
		
Systolic blood pressure	**B = 0.002, 0.001–0.003**	***p* = 0.006**
Diastolic blood pressure	B = 0.001, −0.001–0.003	*p* = 0.453
Neck circumference	B = 0.005, 0.000–0.009	*p* = 0.065
WHR	**B = 0.283, 0.042–0.523**	***p* = 0.022**
SMI	B = 0.004, −0.001–0.008	*p* = 0.148
		
LDL	B = 0.023, −0.005–0.052	*p* = 0.108
Triglycerides	B = 0.026, −0.001–0.054	*p* = 0.055

Statistically significant values are shown in bold. SMI (skeletal muscle mass index), WHR (waist-to-hip ratio), LDL (low-density lipoprotein).

**Table 4:** Multivariable regression analysis of possible CIMT predictors.

	B, 95% C.I. for B	*p* value
Chronological age	B = 0.005, 0.004-0.007	*p* < 0.01
Systolic blood pressure	B = 0.001, 0.000-0.002	*p* = 0.089
		
Chronological age	B = 0.005, 0.004-0.007	*p* < 0.01
WHR	**B = 0.254, 0.056-0.453**	***p* = 0.012**

Statistically significant values are shown in bold. WHR (waist-to-hip ratio).

**Table 5:** Univariable linear regression analysis of possible CIMT Z-score predictors.

	B, 95% C.I. for B	*p* Value
Chronological age	B = 0.008, −0.008–0.024	*p* = 0.348
Vascular age	B = 0.013, −0.013–0.039	*p* = 0.337
Systolic blood pressure	B = 0.011, −0.001–0.023	*p* = 0.068
Diastolic blood pressure	B = 0.013, −0.002–0.029	*p* = 0.089
Neck circumference	B = 0.024, −0.017–0.066	*p* = 0.246
WHR	**B = 2.402, 0.368–4.435**	***p* = 0.021**
SMI	B = 0.029, −0.012–0.069	*p* = 0.163
LDL	B = 0.106, −0.138–0.349	*p* = 0.390
Triglycerides	B = 0.179, −0.052–0.410	*p* = 0.128

Statistically significant values are highlighted. SMI (skeletal muscle mass index), WHR (waist-to-hip ratio), LDL (low-density lipoprotein).

## Data Availability

Available upon reasonable request.

## Supplementary Materials

The following supporting information can be downloaded at: https://www.mdpi.com/article/10.3390/medicina59091512/s1, Table S1: Comparisons of anthropometric and biochemical parameters, and comorbidity frequencies between ‘Vascular age lower then chronological’ and ‘Vascular age higher then chronological’ groups; Table S2: Comparisons of anthropometric and biochemical parameters, and comorbidity frequencies between I–III obesity classes.
