# Supplementary material for: The Interconnection between Carotid Intima–Media Thickness and Obesity: Anthropometric, Clinical and Biochemical Correlations
Source: Medicina (Kaunas). 2023 Aug 23;59(9):1512. doi: 10.3390/medicina59091512 (PMC10534814; doi:10.3390/medicina59091512)
Supplement: Supplementary file 1 [file medicina-59-01512-s001.zip › medicina-2507124-supplementary.pdf]

Variable	Group 1 (n=28)	Group 2 (n=43)	p value
Anthropometric measurements			
BMI (kg/m^2)	43.06±7.66	44.27±8.65	0.549
Weight (kg)	123.09±26.77	126.09±33.52	0.649
Height (m)	168.64±8.32	168.41±10.09	0.918
Body fat percentage (%)	47.33±5.95	44.96±8.18	0.194
Body fat mass (kg)	58.47±16.30	57.45±19.99	0.824
FMI (kg/m^2)	20.56±5.44	20.16±6.05	0.780
Body muscle percentage (%)	50.04±5.69	50.77±10.30	0.737
Body muscle mass (kg)	61.35±14.18	63.51±19.78	0.619
SMI (kg/m^2)	21.36±3.45	22.20±5.63	0.486
Neck circumference (cm)	41.80±4.39	41.34±4.78	0.680
Waist circumference (cm)	127.09±17.66	127.23±22.64	0.977
Hip circumference (cm)	133.71±13.84	135.67±15.44	0.591
WHR	0.95±0.09	0.95±0.10	0.955
Biochemical analysis			
Fasting glucose (mmol/l)	5.71±1.02	5.66±1.10	0.844
Plasma glucose 2 hours after a 75 g oral glucose load (mmol/L)	5.95±1.86	5.42±1.41	0.233
Fasting insulin (mIU/l)	18.04±7.55	17.49±11.32	0.826
HOMA-IR	4.92±2.45	4.75±3.36	0.817
Total cholesterol	4.80±1.05	5.24±1.09	0.097
LDL (mmol/l)	2.84±0.91	3.12±0.91	0.213
HDL (mmol/l)	1.27±0.41	1.30±0.31	0.739
Triglycerides (mmol/l)	1.68±1.05	1.62±00.83	0.758
Comorbidities			
Obesity duration (years)	11.96±6.92	14.28±8.28	0.225
Type 2 diabetes mellitus (%)	21.4	18.6	1.000
Arterial hypertension (%)	67.9	65.1	1.000
Systolic blood pressure (mmHg)	125.79±13.47	137.00±17.88	0.006
Diastolic blood pressure (mmHg)	78.29±8.93	83.40±14.52	0.071
Dyslipidemia (%)	46.4	51.2	0.809

Supplementary Table S1: Comparisons of anthropometric and biochemical parameters, and comorbidity frequencies between 'Vascular age lower then chronological' and 'Vascular age higher then chronological' groups

Group 1 – vascular age lower then chronological

Group 2 – vascular age higher then chronological

Variable	I (n=14)	II (n=30)	III (n=57)	p value
Anthropometric measurements				
BMI (kg/m²)	32.88±2.45	37.47±2.02	49.23±8.56	<0.001#, %
Weight (kg)	90.91±9.91	106.56±12.86	141.59±31.13	<0.001#, %
Height (m)	166.14±5.76	168.48±10.01	169.15±9.31	0.546
Body fat percentage (%)	42.99±4.30	43.65±7.63	47.55±7.00	0.016%
Body fat mass (kg)	39.25±7.43	45.57±6.73	66.19±16.30	<0.001#, %
FMI (kg/m²)	14.17±2.28	16.21±2.98	23.11±4.74	<0.001#, %
Body muscle percentage (%)	54.12±4.10	53.87±6.31	48.76±8.80	0.005%
Body muscle mass (kg)	48.94±3.77	57.99±12.88	68.04±20.26	<0.001#, %
SMI (kg/m²)	17.72±0.79	20.11±2.07	23.73±5.96	<0.001#, %
Neck circumference (cm)	37.61±2.49	39.27±4.54	43.00±4.70	<0.001#, %
Waist circumference (cm)	106.82±9.04	112.93±12.81	136.53±18.59	<0.001#, %
Hip circumference (cm)	115.79±5.30	125.19±7.40	142.62±12.77	<0.001*, #, %
WHR	0.92±0.08	0.92±0.09	0.96±0.10	0.106
Biochemical analysis				
Fasting glucose (mmol/l)	5.44±0.71	5.17±0.93	5.81±1.01	0.013%
Plasma glucose 2 hours after a 75 g oral glucose load (mmol/L)	5.53±1.25	5.17±1.60	5.74±1.75	0.398
Fasting insulin (mIU/l)	12.96±3.92	12.89±6.62	23.58±14.92	<0.001#, %
HOMA-IR	3.12±1.09	3.32±2.15	6.26±3.98	<0.001#, %
Total cholesterol	5.15±1.21	4.99±1.04	4.84±1.08	0.629
LDL (mmol/l)	3.13±1.04	2.94±0.93	2.86±0.82	0.635
HDL (mmol/l)	1.27±0.27	1.24±0.38	1.28±0.31	0.869
Triglycerides (mmol/l)	1.57±0.82	1.69±1.17	1.51±0.77	0.695
Comorbidities				
Obesity duration (years)	8.36±6.97	13.37±9.30	14.11±7.22	0.053
Type 2 diabetes mellitus (%)	7.1	13.3	19.3	0.475
Arterial hypertension (%)	42.9	36.7	75.4	0.001
Systolic blood pressure (mmHg)	127.14±18.11	124.76±18.96	135.19±14.40	0.015%
Diastolic blood pressure (mmHg)	76.50±11.22	80.66±11.86	83.33±13.98	0.198
Dyslipidemia (%)	46.2	46.7	39.3	0.806

Supplementary Table S2: Comparisons of anthropometric and biochemical parameters, and comorbidity frequencies between I-III obesity classes

I - BMI 30.0-34.9 kg/m², II - BMI 35.0-39.9 kg/m², and III - BMI above 40 kg/m²

*statistically significant difference between group 1 and group 2, #statistically significant difference between group 1 and group 3, %statistically significant difference between group 2 and group 3
